# Use of New Media by Turkish Fans in Sport Communication: Facebook and Twitter

**DOI:** 10.2478/v10078-011-0033-x

**Published:** 2011-07-04

**Authors:** Selami Özsoy

**Affiliations:** 1 University of Abant İzzet Baysal, School of Physical Education and Sport, Dep. of Sport Management, Bolu, Turkey

**Keywords:** sports, media, social networks

## Abstract

This research examines the use of Facebook and Twitter, two social networks, for sportive reasons in Turkey.

To this end, the literature was surveyed and a 5 Likert type data collection tool consisting of 21 questions was developed by the researcher based on the expert views. The sample of the research included 460 sport fans who are college students at Abant İzzet Baysal University and Sakarya University.

It was found in the research that 91.7% of the participants had a profile on Facebook and 13.3% had a profile on Twitter. The rate of opening an account on Twitter, which still has no version in Turkish language, was low. It was found that the fans mostly followed the official site of their favorite team on Facebook, got informed about the sports activities through Facebook and learned news, which they did not hear from other sources. It was also ascertained that male fans used social networks for sportive reasons more than female fans did (p<.05).

It is possible to state that social networks such as Facebook and Twitter have become a rapidly-developing alternative medium in sports against traditional media such as newspaper and television.

## Introduction

The Internet, which was introduced into people’s lives within the last 20 years, has changed the mass communication media radically. As Internet has become widespread, personal and institutional web pages (Web 1.0) have shared the effectiveness of classical mass media tools such as newspaper and television. However, social media, which are called Web 2.0 applications, emerged at the early 2000s and have become an alternative to classical media by spreading rapidly. Social media are a rising phenomenon and can be defined as “relating to the sharing of information, experiences and perspectives through community-oriented websites”. Thanks to social media, the geographic walls that divide individuals are crumbling, and new online communities are emerging and growing (Weinburg, 2009).

Social media are a part of what is referred to as Web 2.0. Web 1.0, followed by Web 2.0, was characterized as mostly a one-way communication system. On Web 1.0, a person or company would generate a website and expect the target people to visit the website. Internet sites would offer a limited setting to create and spread a distinctive content personally. Today, the number of people having personal website is quite low. The new Web 2.0 has largely changed the communications medium. In the new media setting, users generate their own content and spread easily at very low costs. Thus, everyone has a voice in the new media setting. Some examples of social media include blogs, forums, message boards, picture and video sharing sites, user-generated sites, wikis and podcast. Each of these tools helps facilitate communication about ideas that users are passionate about, and connects like-minded individuals throughout the world (Weinburg, 2009).

When the expanding process of social media is observed, it is understood that it has grown fast, incomparable to the classical mass communication tools. While radio reached 50 million users after 38 years, TV reached this number of users in 13 years. However, Facebook accrued 100 million users in the first nine months (Steinbach, 2009).

Upon strengthening of the Internet infrastructure and use of mobile tools, opportunities to access social media have diversified. Social media have become more than settings accessed through only desktop or laptop computers. Thanks to the developing information technologies today, access to social media through mobile phones, television and other similar devices has become possible. Mobile phone companies in many countries provide free access to social networks. There are more than 200 million active users currently accessing Facebook through their mobile devices (Facebook Statistics).

Studies conducted by Forrester Research have revealed that young people in the USA spend most of their time on social networks. According to this research, 51% of the ones at the age of 12–17 and 70% of the ones at the age of 17–22 are active users of social networks (koniks.com, accessed February 2011). In another research carried out on college students in Canada, participants reported spending an average of 38.86 minutes on Facebook each day and had between 25 and 1000 Facebook friends (Christofides et al., 2009).

79.6% (17,762) of the Internet users in Turkey use social networking sites. The social network most frequently used by the Internet users is Facebook at a rate of 72.4% (12,770) (comscore.com, 2009). According to a research carried out on college students in Turkey, 70% of the students have Facebook membership. 28% of the Facebook users visit the site once a day while 17% of the users visit the site more than once a day. In each visit to Facebook, 52% of the users spend less than one hour while 39% spend 1–2 hours (Göker et al., 2010).

### Use of social networks for sportive reasons

Sports events, the most attractive subject for people, have become accessible through new generation media as well as the classical settings. Social networks such as Facebook and Twitter, which are spreading day by day, are the most commonly used media for disseminating sports-related news.

The reports show that the online sport demographic is one that is growing at a very fast rate. The people watching sports online are typically people with large disposable incomes (Knight, 2007).

Many sports organizations also use online marketing methods to increase their income. Use of social networks in sports sector for marketing reasons is also becoming common. 51% of active Twitter users follow companies, brands or products on social Networks in USA (Edison Research, 2010).

The effects of social networking sites in advertising the sports activities were researched as a case study in a marathon in USA. The program and promotion of 2009 Cincinnati Flying Pig Marathon was made through favorite social networks such as Facebook, Twitter and Youtube and thus the awareness of this organization by a large mass was increased at almost zero cost. The marathon gained popularity and the interest in the organization was much more than that in the previous years. Several big companies offered sponsorship for the next years’ organization (Schoenstedt and Reau, 2010).

Interactive features of the social media ensure a close connection between the athletes and their fans. The celebrities use social media to establish a direct connection with their fans on Internet. An analysis was carried out in which the contents of the broadcast in the traditional media and social networking sites were compared after a car accident that caused the famous American golf player Tiger Woods a heavy injury. After the accident, Tiger Woods had to disclose his extramarital affairs and got divorced. The analysis suggests that social-media sites are valuable public relations tools that athletes can use to quickly generate support that counteracts perceived negative media framing. Social-media sites also enable fans to enhance perceptions of closeness with athletes as fans interject themselves into athletes’ media narratives (Sanderson, 2010).

### Facebook

Facebook, which is one of the largest social networks in the world, has more than 500 million active users. Facebook was founded in 2004 by a Harvard undergraduate named Mark Zuckerberg. The site was limited to Harvard students in its first month but quickly expanded in response to growing popularity. By the end of 2005, the site had expanded to over 2,000 colleges and 25,000 high schools (Sakuma, 2007).

The Facebook is a social networking site that allows users to create their own virtual profile for the world to see. Users express themselves by putting up their picture, giving basic biographical information, and writing down their interests. Once a user has created their own page, they can interact with other members by visiting their pages and “friending” them. Adding someone as a friend gives a user access to that person’s profile and enables them to interact with them using a variety of applications. The followings are some of the most common applications available to Facebook members.

Facebook site includes an “info” section with descriptive data about the page subject, a section where discussion forums can be created, and a section where photographs and videos can be uploaded, and the page can be linked to other social media channels and Web sites. All pages are configured with a “wall”, a Facebook feature that allows both page creators and Facebook users to write commentary about the page subject, to which other Facebook users can respond. Facebook users also have the ability to indicate that they “like” the page, and when this action is taken, this decision is broadcast to all the user’s Facebook friends. In addition, Facebook provides page creators with data that record how many people each day “like” the page, the number of daily postings on the page, and the number of daily and monthly active users (Sanderson, 2008).

According to Facebook’s data, users spend 700 billion minutes each month on Facebook. There are over 900 million objects that people interact with (pages, groups, events and community pages) and more than 30 billion pieces of content (web links, news stories, blog posts, notes, photo albums, etc.) shared each month. Facebook is used by being translated into 70 languages in the world. 70% of the Facebook users are outside the United States of America (facebook.com, accesed March 2010).

One of the most common reasons for using Facebook was to find a friend when it was founded. A research analyzing the aims of using social networks such as Facebook in high schools revealed that social networks were used mainly to contact with friends and relatives. According to the research, common social network activities included posting photos both for distribution and archival purposes; broadcasting information about social gatherings and sports events; gathering background information about new friends and casual acquaintances; and making contact with lost friends and physically distant acquaintances (Agosto and Abbas, 2010). According to the researches, other main objectives of use of Facebook are as follows: Monitor and audit, video, picture, music and opinion sharing, playing games, organization, politics, e-trading, sexual, and notifications (Sanderson, 2010).

In their very short history, social media have had a profound effect on sport, as many leagues, teams, and athletes have embraced these platforms as a way to talk directly about their lives without having their messages filtered by any marketing or public relations figures.

Framing athletes register for a Facebook account; he or she can create “official” Facebook pages for businesses, brands, products, organizations, artists, bands, or public figures. Facebook requires creators to denote that they are legitimately affiliated with the entity and are authorized to create an official page. These pages mimic a standard Web page, and users have a number of configuration options available to help them design the page.

Social networking sites are used in sports marketing as in other marketing fields. It is stated that the unsold 500 tickets for the football match of the Utah University team in 2008 were sold on Facebook. The authorities contacting 15 thousand fans on Facebook as well as classical press bulletins sold the tickets in a very short period of time. Lessiter, Assistant Sport Director of Utah, said for the usage of social networks: “Press releases are still a very important part of what we do in terms of getting information out, but we realized that in Facebook, Twitter and other social media, we had really powerful ways of getting the word out and letting others spread the word.” (Steinbach, 2010).

In a research carried out on Facebook (sportintelligence, 2007), it was found that the number of fans of the official Facebook sites of famous sport clubs reached millions. According to this research, Turkish club Galatasaray ranked the first with 4.1 million followers, Barcelona ranked the second (3.4 million) and Fenerbahçe ranked third (2.8 million) in the most popular sports club ranking, having an official account on Facebook in 2007. The ranking for the other sports clubs was as follows: Real Madrid (Spain) the fourth (2.7 million), LA Lakers (USA) fifth (2.3 million), Liverpool (UK) sixth (1.9 million), NY Yankees (USA) seventh (1.8 million), Beşiktaş (Turkey) eighth (1.5 million), Boston Red Soxs (USA) ninth (1.3 million), Manchester United (UK) tenth (1.26 million). In an interview published in Sports Intelligence, Ebubekir Kaplan, in charge of marketing and social media of Galatasaray club, answered the question “Why are social networks, especially Facebook, valuable to Galatasaray?” as follows:

We have a lot of fans but only a tiny number of them, relatively, can be in our stadium at the same time. However, all the fans want to meet, both with each other and to stay in touch with the club. Social networking allows this to happen. In Turkey, about 30 percent of the country’s total population is using Facebook, and about 19m adults. The rate of users aged 18 to 44 is 74 %. Turkey is globally the fourth most active country on Facebook, and we can reach out to our fans via Facebook. Players, managers and club officials come and go, but fans are constant; they’re the most important people.

As in Galatasaray case, many sport clubs in Turkey use social networks to contact their fans.

### Twitter

Twitter is perhaps one of the most popular social networking and communication technologies at the present time. It allows individuals to create “microblogs” where they can construct and distribute communicative messages to other. User accounts are linked to a user name preceded by the @ symbol, and messages, or “tweets”, are limited to 140 characters per message. People become connected to others by choosing to follow other users. Each tweet that a person sends is transmitted to each of his or her followers, who can respond by sending a tweet of their own. With the 140 character limit, tweets rely largely on abbreviations. Twitter has become increasingly popular with celebrities, journalist, and sports personalities (Cassing and Sanderson, 2010).

Based on its characteristics, Twitter is often referred to as a microblogging service. Microblogging can best be described as a derivative of blogging, which involves the transfer of news, personal opinion, and ideas in an online setting. Whereas blogging typically involves a dedicated Web site with a main-page focus on expansive content produced by an individual or small group. Twitter uses a much less media-rich interface, where the primary focus is on short bursts of content from a large number of users (Clavio and Kian, 2010).

Although Twitter was introduced after Facebook, it has grown rapidly. While Twitter had 1.22 million visitors in April 2008, it reached 17.10 billion visitors in April 2009 with an increase of 1298%. On the other hand, while Facebook had 22.48 million visitors in April 2008, it reached 71.29 million visitors with an increase of 217% (Johnson, 2009). Only 5% of the population in the USA was informed about Twitter in 2008 but the rate increased to 87% in 2010. It was found that 17 million people have an account on Twitter in the USA. While the rate of Twitter users was 7%, the rate of Facebook users was 41% in the USA (Edison Research, 2010).

According to the data published by Twitter in 2011, the fifth anniversary of its foundation, nearly 1 billion tweets are sent weekly. It took 3 years, 2 months and 1 day to reach 1 billion messages sent since its foundation. In March 2010, the users sent approximately 50 million messages in a day. In March 2011, the number of tweets sent in a day reached 140 million. Nearly 460,000 new accounts are opened in a day on Twitter. The number of Twitter users on mobile phones increased by 182% compared to the one in 2010 (twitter release, accessed March 2011). Nearly two third of 17 million Twitter users in the USA access social networks through mobile phones (Edison Research, 2010).

Social media and Web 2.0 have also transformed the interaction between sport fans and their sport heroes. In Web 1.0, fans could visit a team, league, or athlete Web site and peruse the content posted on the site. The only interaction available to them was through e-mail or, if the Web site had one, a message board.

With Web 2.0, the situation has changed. Teams, leagues, and athletes area embracing social media and using them to bring fans closer to the game (Pegoraro, 2010).

The social-networking phenomenon of Twitter has made considerable inroads into the sport communication landscape since its introduction in 2006. One of the fastest growing Web 2.0 applications in the new-media marketplace, Twitter combines several unique aspects of communication, which makes it attractive to both sports fans and sport organizations (Clavio and Kian, 2010). Twitter has brought fans closer to their sport heroes because it allows athletes to communicate as openly and honestly as they wish without any third-party mediation.

Twitter offers a setting ensuring direct communication between fans, athletes, trainers and sports reporters.

The NBA had accrued over 1.93 million followers on Twitter, with the NFL (1.63 million), the Los Angeles Lakers (1.56 million), and the Orlando Magic (1.01 million) also reaching seven figures in Twitter followers in June 2010.

Twitter accounts of many world-famous athletes have millions of followers. For example, Shaquille O’Neil basketball player for Boston Celtics has 3,5 million, Kaka in Real Madrid has 3 million, Cyclist Lance Armstrong 2,8 million, Skateboarder Tony Hawk 2,4 million, Christiano Ronaldo in Real Madrid 2 million, Tennis player Serena Williams 2 million, Dwight Howard basketball player for Orlando Magic 1.9 million, Chad OchaCinco Johnson in Cincinati Bengals 1.8 million, Skateboarder Ryan Sheckler 1,8 million, Paul Pierce in Boston Celtic has 1.8 million followers (tweeting-athletes, accessed March 2011).

Use of Twitter in sport communication has been a subject to several researches. These researches have examined the use of Twitter in sports media and the contents of the sports-related messages (Debatin et al. 2009, Sheffer & Schultz 2010a; Sheffer & Schultz 2010b; Sanderson 2010; Pegoraro 2010; Clavio & Kian 2010, Kassing & Sanderson 2010, Johnson 2010).

In research on the effects of Twitter on sports journalism, it was found that sports journalists of different demographic structures perceived new technologies in different ways. Younger and broadcast journalists were more likely to see Twitter as having stand-alone value and use it in forward-thinking ways. Older and print journalists were more likely to use Twitter for traditional purposes such as promoting printed work on other platforms (Sheffer & Schultz, 2010a). In a study which examined how sports journalists used Twitter for their profession, the journalists indicated that they used Twitter most often for breaking news, followed closely by promoting work on traditional media outlets, and then connecting with audiences. Sports journalists indicated that they seldom used Twitter to express their own personal opinion (Sheffer & Schultz, 2010b).

Another research which examined the contents of Twitter messages of the athletes in USA revealed that the athletes post messages about their daily routines, weather, party reference, charity work and family life, professional training, traveling, game preparations, promoting products about their personal lives as well as other games in their league, college or minor-league games of the same sport and equipment about their sportive lives. The athletes also post messages about TV shows, movie, musician or group, actor or actress politician and famous landmark about pop culture (Pegoraro, 2010).

In a research which analyzed the followers of a retired female athlete, the followers indicated that they followed the athlete for her competency in her field the most. 89% of the 216 participants of the questionnaire stated that they visited the Twitter page at least once a day. The rate of the male participants stating that they followed the athlete because they found her physically attractive was found to be meaningfully more than the female participants did (Clavio & Kian, 2010).

In a research which examined the use of Twitter by professional athletes to contact with their fans and other players, the tweets were gathered under six categories such as interactivity, diversion, information sharing, content, promotional, and fanship. Many of the tweets fell into the interactivity category (34%) (Hambrick et al., 2010).

Facebook and Twitter both function as social networking services. However, Facebook as a social network is much flexible and versatile. You can upload pictures, videos, games, and apps to profile; embed videos from some sites; and post calendar events. Twitter, at first glance, only allows for text, more text, and even more text with links. Also, Twitter is a microblogging service while Facebook has many facets including a microblogging component.

A major difference between Facebook and Twitter emerges in their methods of communication. Facebook is meant to be more passive. In contrast, Twitter seems a much more active form of social communication in which the way you talk to people on the social network emerges as much conversational. Twitter has been linked to a giant party where you know no one but wish to make many friends. In contrast, Facebook would be a wedding reception filled with family and friends. When looking at these two tools, one issue comes up quite frequently the issue of privacy. Privacy seems paramount to the users of Facebook, but Twitter users tend to embrace the feeling that everything is public. Simply look at this difference in the two services: Facebook gives you friends, while Twitter gives you followers (Taghmeier, 2010).

### Twitter in Turkey

Despite its fast growing graphic, Twitter has fallen behind Facebook in Turkey since it has no Turkish version. However, that many statesmen such as the President and Prime Minister, politicians and athletes generate profiles and their tweets come to the agenda of the media increases the attractiveness of this social network.

Many clubs, athletes, technical staff and managers in Turkey have been opening an account on Twitter recently and these accounts are followed by fans whose numbers increase day by day. Galatasaray and Trabzonspor, Sport Toto Super League clubs in football which is the most common sport in Turkey, are the two of these clubs. Trabzonspor announced the opening of its official Twitter site in its official Internet site in May 2010 as follows:

“Our club which has fans all over the world has planned to contact with our fans in a wider and updated setting. Our fans will have the opportunity to broadcast on their personal websites the news through the links provided below the news in our official website” (Trabzonspor.org, accessed March, 2011).

It is known that some athletes have problems because of their tweets on Twitter. Galatasaray football player Colin Kazım - Richards had to face the reactions of the opponent fans because of his sarcastic tweets as “BJK, 8JK ha ha” before the Beşiktaş derby when he was a player in Fenerbahçe (Hurriyet, 2009). That Aykur Kocaman, Technical Director of Fenerbahçe, criticized Gökhan Gönül, football player at Fenerbahçe, for his discussion with the Galatasaray fans on Twitter, opened the door for a new discussion in Turkey. Upon this, the internet site of the Hürriyet newspaper introduced a questionnaire on **“**Should athletes use social networking sites, especially Twitter?”. According to the results of the questionnaire, the most voted view was “Athletes should be on such websites but should not give wrong messages” (Hurriyet, 2011). Moreover, athletes sometimes had to face penalties for their statements on Twitter. Mark Cuban, the owner of the Dallas Mavericks, was fined $25,000 for criticizing the referees after a game on Twitter.

### Twitter piracy

Some celebrities, including athletes, in Turkey face some difficulties due to the accounts opened in their names by others on social networking sites. Use of unreal news on these accounts by print and visual media leaves these celebrities in the lurch.

Showman Cem Yılmaz said for the accounts opened in his name: “People, who are suffering from the illness of opening an account for somebody else’s name, which can be called as “electronic insanity”, that I considered as the illness of the age, give statements and make announcements on behalf of me. I have no virtual account or initiative on popular networking sites of the Internet world (Facebook-Twitter)”.

Emre Belözoğlu, football player at Fenerbahçe, and Arda Turan, football player at Galatasaray, are two of the football players having problems because of the Twitter accounts opened on behalf of them. Before his transfer to Fenerbahçe team, Emre Belözoğlu, had to declare that neither his wife nor he had membership to any social networks after the news on press as “He heralds transfer to Fenerbahçe fans through Twitter”. Arda Turan also had to declare he had no membership to any social networks by stating that the media broadcasted news from the accounts which did not belong to him (sporyolu.com, 2010).

### Purpose

The purpose of this research is to examine the use of Facebook and Twitter, two of the most favorite social networks, by fans in Turkey to follow the sport events.

The following hypotheses were tested in the research:

H1: Fans use Facebook, among social networks, for sportive reasons.
H2: Fans use Twitter, among social networks, for sportive reasons.
H3: Use of Facebook and Twitter by fans differs according to gender.
H4: Use of Facebook and Twitter by fans differs according to age groups.
H5: Use of Facebook and Twitter by fans differs according to level of income.
H6: Use of Facebook and Twitter by fans differs according to their physical exercise habits.

## Material and Methods

620 students studying at Abant İzzet Baysal University and Sakarya University participated in the research. 460 participants who declared to be a fan of a sports club were the sample of the research.

A 5 Likert type data collection tool consisting of 21 questions was developed by the researcher in line with the literature survey and comments of two experts and user opinions. The data collection tool was applied to the students, who were randomly selected at School of Physical Education and Sports, Faculty of Arts and Science and Vocational School at the two universities in October 2010.

The obtained data, percentage values and independent samples were tested by the *t*-test and one-way variance analysis (ANOVA). The reliability coefficient was found to be α=.9151 in the reliability analysis of the 21 items.

21 questions in the data collection tool used in the research consisted of the subjects categorized under 3 main groups. The first group included questions about the participants’ following Internet and blogs on Internet; the second included those about Facebook and the third included those about Twitter. Factor analysis was applied to the data collection tool and the 21 items were reduced to three factors. These factors validating the three question groups built at the beginning of the research were labeled as “Internet and blog use”, “Facebook” and “Twitter”. The three items obtained in the factor analysis explain 70.7% of the total variance. Kaiser-Meyer-Olkin measure of Sampling Adequacy 0.915 and Bartlett’s Test of Sphericity were found to be meaningful at .000 level.

The interviews with the social networks users and the message sharing followed by the researcher on his Facebook and Twitter accounts were used as qualitative data supporting the findings.

## Results

### Personal Information

46.5% of 420 college student fans, the samples of the research, are female while 53.5% are male. 78.5% of the participants study at Abant İzzet Baysal University while 21.5% study at Sakarya University. 48.9% of the students study at School of Physical Education and Sport and the others study at different departments (27.4% at Faculty of Education, 14.1% at Bolu Vocational School and 9.6% at Faculty of Arts and Science). 35% of the participants are freshmen, 27.2% are sophomores, 20% are juniors and 17.8% are seniors.

According to their age ranges, 28.9% are between 17–19, 43.5% are between 20–22, 19.1% are between 23–25, and 8.5% are over or at the age of 26.

According to their families’ level of monthly income, 13.5% of the participants have an income less than 600 TL (approx. $380), 40.4% between 601–1200 TL (approx. $380–$770), 29.8% between 1200–1800 TL (approx. $770–$1150), and 16.1% over 1801 TL (approx. $1150).

17.2% of the participants stated that they were active in sports while 82.8% stated they were not. 91.7% of the participants stated that they generated a profile on Facebook, 13.3% on Twitter, 8.5% on Myspace, and 0.9% on Friendfeed. 7.2% of the participants are not a member of any social networks.

Although the fans who participated in the research follow Facebook frequently (x=4.50), the average of the ones who stated that they followed Twitter was lower (x=1.38).

The average of the fans following sport news on Internet sites was high (x=4.05). The fans frequently follow the official profile of their favorite team on Facebook (x=3.45). The fans stated that they were informed about the sport activities through Facebook (x=3.33). The fans stated that they mostly learned on Facebook about the sport news they did not hear from other sources (x=3.31) (Table 2).

The fans also stated that they mostly shared the sport videos on Facebook (x=3.17). Video sharing is one of the most commonly used functions of Facebook. The fans post the goals shot in the matches on their Facebook profiles by giving links to video sharing sites like Youtube. It is observed that mostly the goal videos and interesting moments in matches are shared by the fans following the matches on Sport Toto Football Super League in Turkey.

After the league matches in football, the fans make comments on Facebook about the status of their favorite teams (x=2.99). Members share humorous messages with their team’s fans or opponent fans after matches on Facebook, which is popular for giving opportunity to share opinions and ideas among members.

After the first derby match in Türk Telekom Arena, the newly built stadium of Galatasaray, where Fenerbahçe beat Galatasaray 2-1, Fenerbahçe fans posted various messages on their profiles. Some of them are as follows:

- A Galatasaray fan has the right to glory for 75 minutes the most... The remaining minutes are a bottle of rakı...
- Not again, even in this stadium. TOKI should build a new one so we can beat them there, too.
- 3D: Derby, Decibel, Drama
- 3D: Déja vu, Déja vu, Déja vu... Enough is enough.
- Fenerbahçe registered Türk Telekom Arena officially. Enjoy it!
- The stadium changes, the players change but the winner is the same.

The fans stated that they followed sport blogs on Internet sites less than they did on Facebook (x=3.00). They stated that they mostly followed their favorite athletes (x=1.28) and the official account of their favorite club (x=1.25) on Twitter, where the following rate is low (Table 2).

### Use of Facebook and Twitter by fans according to gender

The independent samples *t*-test carried out to find out the differences in use of social networking sites by fans according to gender revealed that male fans followed social networks for sportive reasons more frequently than female fans did. As a result of the test, meaningful differences were found for the 12 items according to gender (p<.05).

Accordingly, men follow sport news (*t*=13.5, p<.05) and sport blogs (*t*=8.29, p<.05) more frequently. Male fans write blogs more on sport related Internet sites (*t*=6.80, p<.05). Male fans follow Facebook (*t*=4.95, p<.05) more frequently than female fans do. There was a meaningful difference (*t*=6.15, p<.05) in male fans in following the favorite team’s official site on Facebook (*t*=6.17, p<.05) and sharing sport videos compared to female fans. It was found that male fans made friends with athletes (*t*=4.11, p<.05) and other fans (*t*=5.31, p<.05) through Facebook more than female fans did. Male fans were found to post more comments on sport (*t*=7.54, p<.05) and messages on Facebook after their favorite team won (*t*=4.15, p<.05). Through Facebook, male fans are more informed about the sport activities (*t*=4.17, p<.05). Male fans broadcast sport news on Internet sites through their Facebook accounts more than female fans do (*t*=4.73, p<.05).

### Use of Facebook and Twitter by fans according to their physical exercise habits

Meaningful differences were found in the *t* test analysis made to analyze the use of social networks by the college student fans according to their physical exercise habits. According to the test results, fans who are active in physical exercises follow sports news (*t*=5.20, p<.05) and sports blogs (*t*=4.68, p<.05) and write blogs in sport sites (*t*=4.58, p<.05) more than the fans who are not. Also, the fans who do physical exercise make friends with athletes through Facebook (*t*=4.29, p<.05) more than the ones who do not.

### Use of Facebook and Twitter by fans according to level of income

No meaningful difference was found in the One Way Variance Analysis (ANOVA) made on the four groups according to the variable of level of family income in use of social networks by the sample fans (p< 0.05).

### Use of Facebook and Twitter by fans according to age groups

In the One Way Variance Analysis (ANOVA) made according to age groups in use of social networks by the sample fans, differences were found in the items of following sport news, following sport blogs and writing blogs.

The Tukey test made to define the groups having differences revealed that the fans at the 17–19 age group followed the sport news on Internet more frequently than the ones at the age groups 20–22, 23–25 and over 25 did F (3,455)=9.415, p<.05.

It was found that the 23–25 age group followed the sport blogs on Internet more frequently than the 17–19 age group did F (3,452)=3.372, p<.05.

The fans at the 23–25 age group write more blogs on sport sites than the ones at the 17–19 age group do F (3,447)=3.146, p<.05. It was also found that the 23–25 age group shared more sport videos on Internet than the 17–19 age group did F (3,454)=3.146, p<.05.

## Discussion and Conclusion

This research revealed that fans studying at a university in Turkey follow sport news frequently on Internet (x=4.05). Internet has reached a large number of users in Turkey as in the world. According to a measurement in April 2009, there are 17,762 million Internet users in Turkey. In terms of largest online population in Europe, Turkey was the seventh largest with 17,762 thousand million visitors while Germany’s online audience was the largest with 40 million visitors, followed by the U.K. (36,820 million visitors), France (36.3 million visitors), Russia Federation (31,303), Italy (21,230) and Spain (18,636). Internet users in Turkey were also found to be the most engaged users in Europe, spending an average 32 hours and viewing an average 3,044 pages of content per month (comscore.com, 2009).

The fans in the research stated that they followed sport news on Facebook as frequently as they followed the sport news on Internet. According to the data obtained, 91.7% of the participants followed Facebook, the most common social networking site in Turkey. This rate is higher than the one measured in another research examining the prevalence of Facebook. According to another research on college students in Turkey, 70% of the participants have a Facebook membership (Göker et al., 2010).

The necessity to access new media through only computer has disappeared thanks to technological innovations such as 3G and 4G. Today, easy access to social networking sites has become possible through mobile phones and palmtop computers (PDA) which are commonly used, which ensured access of fans to news about sport events and match scores regardless of time and place. New media are preferred more due to their low cost. That GSM operators, providing mobile communication service in Turkey, ensure free access to social networks, although limited, is one of the reasons for the increase in prevalence.

In this research, the participants stated that they mostly shared sport videos on Facebook (x=3.17). One of the reasons for use of social networks for sportive reasons is that they give opportunity to follow instantly changing circumstances such as goals in a match and scores. The users also have the opportunity to watch the videos as much as and when they like.

In the research, the rate of following Twitter was found to be lower than that of Facebook (% 13.3). In a research conducted in the USA in 2010, the awareness rate of Twitter (87%) and Facebook (88%) was found to be equal. Still, Twitter has 17 million users in the USA and the rate of this number to the population is 7%. On the other hand, the rate of Facebook users in the USA is 41% (Edison Research, 2010). In another research in the USA (Clavio & Kian, 2010: 492), 89% of the participants stated that they visited their Twitter page at least once a day. Low level of use of Twitter in Turkey, which is a common social networking site in the world, can be related to nonexistence of the Turkish version of the site.

Social media, such as Facebook and Twitter, are also effectively used by sport journalists. This fact is supported by many academic studies (Sheffer & Schultz, 2010b). News resources of the media such as clubs, managers and athletes have started to release statements through social media. Fans have the opportunity to learn the events at the same time as the sport journalists by following the social media they follow. Thus, the rate of following new media increases. It may be envisaged that the effectiveness of traditional mass media tools will go down and the classical media failing to renew themselves will fall behind in the race in the future.

This study carried out on the fans studying at a university revealed that there were meaningful differences in status of following sport events on social networks by the participants in terms of their gender, age, physical exercise habits and level of income (p<.05).

It was found that male fans followed social networks for sportive communication more frequently than female fans did, according to distribution by gender. The obtained data are parallel to the results of the study carried out in the USA. According to the results of the research conducted by Nielsen / NetRatings Net Wiew 58.18% of the online sport audiences are male while 41.82% are female (Loechner, 2007). That men follow sports more than women do in Turkey can be explained by the fact that the most popular sport is football and football is mostly followed by men.

Although there is almost an equal distribution among the groups considering the level of family income of the participants, no difference was observed in use of social networks, which results in the fact that social networks are used at the same frequency whatever the level of income is. Moreover, free access to social networks provided by GSM operators, which offer mobile communication service in Turkey, is one of the reasons for the increase in prevalence.

It was found that the fans were informed about the sport activities in their community through Facebook and learnt news on facebook which are not available on other resources. As in many countries in the world, the media mainly release football news in Turkey. Branches other than football and local amateur sport activities are not commonly broadcasted on mass media. For this reason, sport fans share events about amateur branches on new media settings such as social networks. Thus, sports news which is not given in classical media like newspaper and television is circulated on social networks. It is observed that national or local sport activity organizers open pages on Facebook to announce many local and national sport activities. Announcement of local organizations, particularly with minor target group, through social networks is becoming common.

In the analysis made according to the ages of the participants, no meaningful difference was found in the use of Facebook and Twitter. There was difference only in the items of following sport news on Internet, following sport blogs and blog writing. Despite the fact that the sample college students were at a narrow age range, it is possible to say that this generation, that can also be called young, use social networks regardless of age.

In conclusion, sport news reaches the target group through not only traditional mass communication tools like newspaper and television but also internet-based new media today. Sports fans using communication technologies now learn the sportive events such as match scores, statements of athletes and managers through new media that are more practical, cheaper and faster. It is possible to say that social networks are more commonly becoming an alternative to classical information obtaining methods for the fans. The research revealed that the fans, the sample of the research, followed Facebook, one of the social networking sites, and this site played an effective role in transmitting sport news. However, the fact that the sample of the research consisted of college students, whose aptness to and interest in communication technologies were expected to be high, must be taken into consideration when interpreting the results.

## Figures and Tables

**Table 1:** The social networks on which the participants generated a profile

**Site**	**Yes**		**No**		**Total**	
	
	n	%	n	%	n	%
Facebook	422	91.7	38	8.3	460	100
Twitter	61	13.3	399	86.7	460	100
Myspace	39	8.5	421	91.5	460	100
Friendfeed	4	.9	456	99.1	460	100

**Table 2:** Use of Facebook and Twitter by the participants

**ITEMS**		Average
1.	I follow Facebook.	4.50
2.	I follow sport news on Internet.	4.05
3.	I follow my favorite team’s official website on Facebook.	3.45
4.	I am informed about sport activities in my community through Facebook.	3.33
5.	I am informed on Facebook about the news not available on other resources.	3.31
6.	I share sport videos on Facebook.	3.17
7.	I follow sport blogs on Internet.	3.00
8.	I post a message on Facebook after my team wins.	2.99
9.	I post comments on sport on Facebook.	2.52
10.	I broadcast sport news on Internet sites through Facebook.	2.46
11.	I make friends with athletes through Facebook	2.11
12.	I make friends with other fans through Facebook.	2.07
13.	I write blogs on sport sites on Internet.	1.98
14.	I follow Twitter.	1.38
15.	I follow my favorite athletes through Twitter.	1.28
16.	I follow my favorite team’s official site on Twitter.	1.25
17.	I am informed through Twitter about the news not available on other resources.	1.25
18.	I follow fan groups of my favorite team on Twitter.	1.23
19.	I am informed about the sport activities in my community through Twitter.	1.23
20.	I post sport tweets on Twitter.	1.22
21.	I broadcast the sport news on Internet sites through Twitter.	1.19
